# Lactate Dehydrogenase Superfamily in Rice and *Arabidopsis*: Understanding the Molecular Evolution and Structural Diversity

**DOI:** 10.3390/ijms24065900

**Published:** 2023-03-21

**Authors:** Yajnaseni Chatterjee, Bidisha Bhowal, Kapuganti Jagadis Gupta, Ashwani Pareek, Sneh Lata Singla-Pareek

**Affiliations:** 1 Plant Stress Biology Group, International Centre for Genetic Engineering and Biotechnology (ICGEB), Aruna Asaf Ali Marg, New Delhi 110067, India; 2 National Institute for Plant Genome Research, New Delhi 110067, India; 3 Stress Physiology and Molecular Biology Laboratory, School of Life Sciences, Jawaharlal Nehru University, New Delhi 110067, India

**Keywords:** L-lactate dehydrogenase, malate dehydrogenases, docking score, abiotic stress, superfamily

## Abstract

Lactate/malate dehydrogenases (Ldh/Maldh) are ubiquitous enzymes involved in the central metabolic pathway of plants and animals. The role of malate dehydrogenases in the plant system is very well documented. However, the role of its homolog L-lactate dehydrogenases still remains elusive. Though its occurrence is experimentally proven in a few plant species, not much is known about its role in rice. Therefore, a comprehensive genome-wide in silico investigation was carried out to identify all Ldh genes in model plants, rice and *Arabidopsis*, which revealed Ldh to be a multigene family encoding multiple proteins. Publicly available data suggest its role in a wide range of abiotic stresses such as anoxia, salinity, heat, submergence, cold and heavy metal stress, as also confirmed by our qRT-PCR analysis, especially in salinity and heavy metal mediated stresses. A detailed protein modelling and docking analysis using Schrodinger Suite reveals the presence of three putatively functional L-lactate dehydrogenases in rice, namely OsLdh3, OsLdh7 and OsLdh9. The analysis also highlights the important role of Ser-219, Gly-220 and His-251 in the active site geometry of OsLdh3, OsLdh7 and OsLdh9, respectively. In fact, these three genes have also been found to be highly upregulated under salinity, hypoxia and heavy metal mediated stresses in rice.

## 1. Introduction

Lactate dehydrogenases (Ldh) and malate dehydrogenases (Maldh) are members of the homologous superfamily of 2-ketoacid NAD dependent dehydrogenases that catalyse the conversion of 2-hydroxyacids to their corresponding 2-ketoacids [1]. Both the enzymes share a common evolutionary origin indicated by their structural similarities, displaying a common protein fold and similar catalytic mechanisms [2].

**Figure 1:**
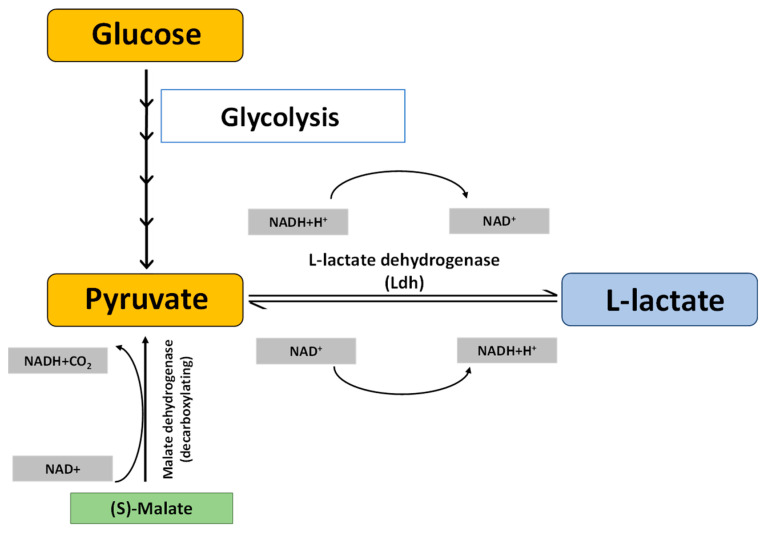
**Schematic representation of L-lactate metabolism:** Pyruvate, the end-product of glycolysis, is converted to L-lactate by the L-lactate dehydrogenase (Ldh) enzyme. This reversible reaction converts NAD^+^ to NADH+H^+^ and vice versa. Pyruvate is also formed by the decarboxylation of (S)-Malate, catalysed by the Malate dehydrogenase (Maldh) (decarboxylating) enzyme with the concomitant release of NADH and CO_2_, which is then converted to L-lactate by the Ldh enzyme.

Maldh (E.C. 1.1.1.37) is a ubiquitous enzyme catalysing the reversible conversion of oxaloacetate (OAA) into malate. It serves a crucial role in various other significant metabolic pathways, such as the amino acid biosynthetic pathway, in glyoxalate bypass, gluconeogenesis, and in facilitating the exchange of metabolites across sub-cellular compartments, the Kreb’s cycle being one of the most important of them. Eukaryotes possess multiple forms of Maldh involved in different cellular processes, differing in their specificity for NAD and NADP [3]. All Maldhs are NAD-dependent except chloroplastic Maldh, which is NADP dependent [4]. Maldhs have been extensively studied in plants [5,6,7], especially the role of NADP dependent plastid localized Maldh. NADP-Maldh (E.C 1.1.1.82) is found both in C3 and C4 plants. In C4 plants, it is responsible for the primary fixation and transfer of carbon dioxide, while in C3 plants it is involved in chloroplast shuttle mechanisms which may be helpful in transporting redox power [3]. In addition, malate dehydrogenase (decarboxylating) or NAD-ME (E.C. 1.1.1.39) catalyses the conversion of malate to pyruvate using NAD as a cofactor and concomitantly releasing carbon dioxide (Figure 1). Ldhs (E.C. 1.1.1.27), on the other hand, operate at the final stages of glucose metabolism (Figure 1), preferably during anaerobic glycolysis. They catalyse the reversible conversion of L-lactate to pyruvate with the concomitant reduction of NAD+ to NADH, and vice versa [8]. Ldhs were earlier believed to be common in bacteria and animal tissues, with little or no detectable amount of the enzymes in plants. James and Cragg (1943) [9] were reportedly the first to observe Ldh activity in higher plant tissues. The first few reports on Ldh from plants found it to be present in tissues such as roots, seedlings and potato tubers, with its function assumed to be linked to anaerobic metabolism [10,11,12,13]. The presence of Ldh was also reported in the leaves of lettuce plants [14]. Thereafter, Ldhs have been found to occur in all green plants ranging from flowering plants to mosses, with varying tissue distribution and enzyme activity levels [15]. The role of plant Ldh in hypoxia and/or anoxia became well established in the subsequent studies on different crop plants [16,17,18]. 

*Arabidopsis* Ldh1 is to date the only plant Ldh enzyme reported to be expressed under stresses other than hypoxia, such as drought, cold and mechanical wounding [19]. Since then, there have been very few studies on Ldhs in plants. This encouraged us to carry out a detailed genome-wide analysis of the Ldhs in plants, and explore their functional role in plant physiology and stress response. Thus, we carried out a comprehensive pan-genome study to identify the genes encoding Ldh in rice and *Arabidopsis*, and explore their role in major abiotic stresses such as heat, cold, salinity, and drought, as well as submergence, since its abundance in stresses other than hypoxia/anoxia is largely undetermined. Interestingly, we found *Ldh* to be a multigene family consisting of both lactate and malate dehydrogenases, differentially regulated in various tissues, stresses and hormonal treatments. Among the various abiotic stresses, seven out of twelve Ldh genes showed significant up-regulation under salinity stress, implying its role in imparting salt stress tolerance in plants. Another intriguing aspect that we have highlighted in this study is the substrate binding affinity of Ldh for malate and/or lactate using structural and docking analysis, especially during the reversible reaction, as both the substrates are present in the cellular milieu.

## 2. Results

### 2.1. Identification and Evolutionary Analysis of Ldh Genes across Plant Species

**Figure 2:**
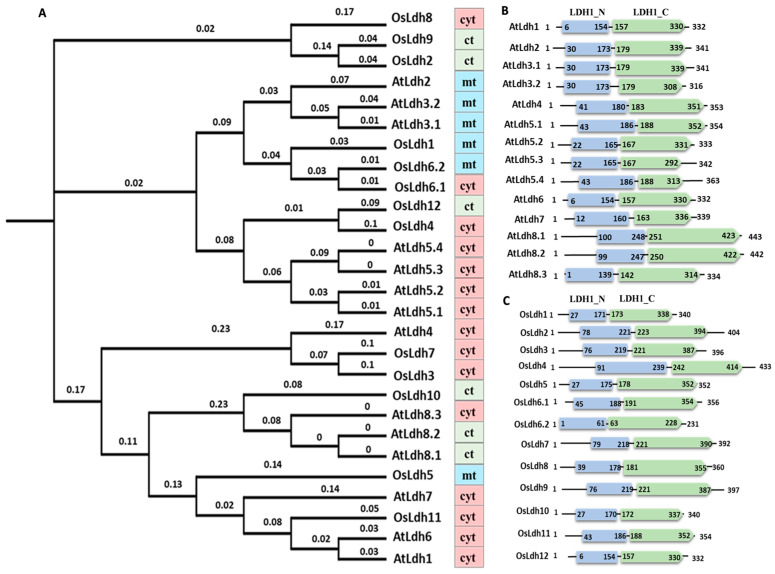
**Evolutionary analysis and domain organisation of lactate/malate dehydrogenases in model plants, rice and *Arabidopsis.*** (**A**) Phylogenetic tree depicting evolutionary relationships among Ldh proteins in rice and *Arabidopsis*, generated using ClustalΩ. The colour strip denotes the predicted sub-cellular localization of the respective proteins (pink—cytoplasm (cyt), blue—mitochondria (mt), green—chloroplast (ct). The branch lengths indicate evolutionary distance between two nodes. (**B**) Domain organisation of lactate/malate dehydrogenases in *Arabidopsis*. (**C**) Domain organisation of lactate/malate dehydrogenases in rice. The figures show the presence of LDH1_N (blue) and LDH1_C (green) domains characteristic of Ldh proteins. Domains were analysed using SMART database. Position and number of domains are schematically represented along with the length of the protein and are not to scale.

The Ldh/Maldh superfamily consists of two characteristic catalytic domains, namely LDH1_N (pfam00056) and LDH1_C (pfam02866). Based on these two domains, a thorough search against the TAIR and RGAP database led to the identification of twelve genes in rice (*Oryza sativa*) and eight genes in *Arabidopsis thaliana*, suggesting that, like other gene families, Ldh genes also encode multiple proteins across plant species. The chromosomal localization, 5′-3′ coordinates, CDS length, and protein length, along with their physico-chemical characteristics, have been listed in Table 1. The proteins mostly range from 300 to 400 amino acids in length, with an average molecular weight of 38 kDa and an average iso-electric point (pI) of 7.06. Since both malate and lactate dehydrogenase consist of the same domains and there exists no uniform nomenclature for proteins possessing these domains, we have hereafter assigned the label ‘Ldh’ for denoting the genes encoding lactate/malate dehydrogenases. The prefixes “At” or “Os” have been added before “Ldh”, followed by Arabic numbers for nomenclature. The identified members were then scanned in the Prosite database. We found two out of the twelve members (OsLdh3, OsLdh7) in rice and one (AtLdh4) out of eight members in Arabidopsis to have the L-lactate dehydrogenase enzyme specific active site signature motif (PS00064), while the rest of the proteins possessed the active site signature of malate dehydrogenase enzyme (PS00068). In order to study the distribution of the genes and their evolutionary divergence in monocots and dicots, protein sequences from *Arabidopsis* and rice were aligned. Based on the sequence similarity obtained via Neighbor-Joining method, a phylogenetic tree was constructed (Figure 2A). It has been found that the majority of the proteins are predicted to be localized in the cytoplasm followed by chloroplasts and mitochondria. The functionally active AtLdh4 has been predicted to be localized in the cytoplasm. OsLdh3 and OsLdh7, which clustered with AtLdh4, are found to localize in the cytoplasm.

Further, the domain characterization of the Ldh proteins in the model plant species showed the functional domains, LDH1_N and LDH1_C characteristic of its catalytic activity to be conserved in the species and placed 2-3 amino acids apart in all the proteins. No additional domain is present in any of the proteins (Figure 2B,C).

### 2.2. Exon/Intron Organization and Protein Motif Analysis of the Rice and Arabidopsis Ldh Genes and Proteins

**Figure 3:**
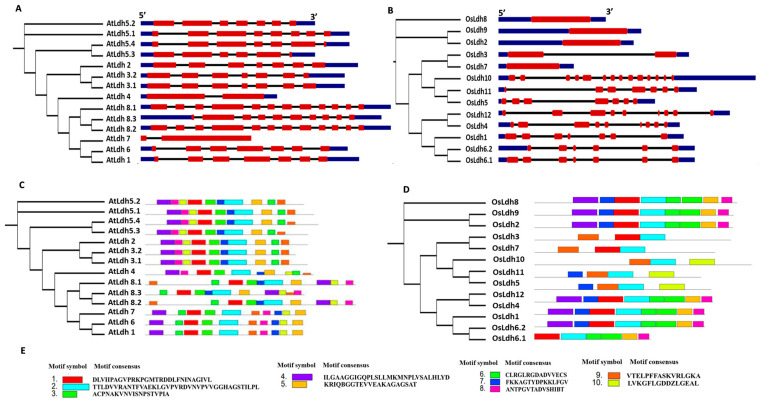
**Schematic representation of exon–intron architecture of genes encoding lactate/malate dehydrogenases in model plants *Arabidopsis* and rice.** The exon–intron structures of the genes encoding lactate/malate dehydrogenases in (**A**) *Arabidopsis* and (**B**) rice were made using the Gene Structure Display Server (GSDS) tool. The exons and introns have been depicted in red and black, respectively, and the UTR regions are depicted in blue. The length of exon and introns are not to scale. Motif organization in (**C**) *Arabidopsis* and (**D**) rice Ldh proteins analysed using MEME Suite. (**E**) List of consensus motifs identified in rice and *Arabidopsis* using MEME Suite.

The exon/intron organization of the Ldh genes was analysed in *Arabidopsis* (Figure 3A) and rice (Figure 3B) to understand the structure of the respective genes. In rice, *OsLdh2*, *OsLdh7*, *OsLdh8* and *OsLdh9* were found to be intron-less, whereas there were no such genes in *Arabidopsis*. *AtLdh4* had a similar exon/intron organization to that of *OsLdh3*, both having two exons and one intron. The length of introns differed, though. In *OsLdh3*, intron length was around 2 kb, while that of *AtLdh4* was only few bps. *OsLdh10* had 14 exons and 13 introns, while *AtLdh8.1*, *AtLdh8.2* and *AtLdh8.3* had 12, 10 and 12 exons and 11, 9 and 11 introns, respectively. *AtLdh2*, *AtLdh3* and their isoforms had the same number of exons (seven) and introns (six) as those of *OsLdh6* and *OsLdh1*. Protein motif analysis revealed 10 signature motif sequences to be conserved in the AtLdh and OsLdh proteins. However, the motif site arrangement varied in the different members of the same species. In *Arabidopsis*, AtLdh2, AtLdh3.1/3.2, AtLdh4 and AtLdh5.1/5.2/5.3/5.4 showed similar motif arrangement, while, in rice, OsLdh3 and OsLdh7 shared similar motif arrangement, dissimilar from the other members of the family (Figure 3C–E).

### 2.3. Tissue-Specific Variations and Stress-Mediated Expression Profiling of Ldh Genes 

**Figure 4:**
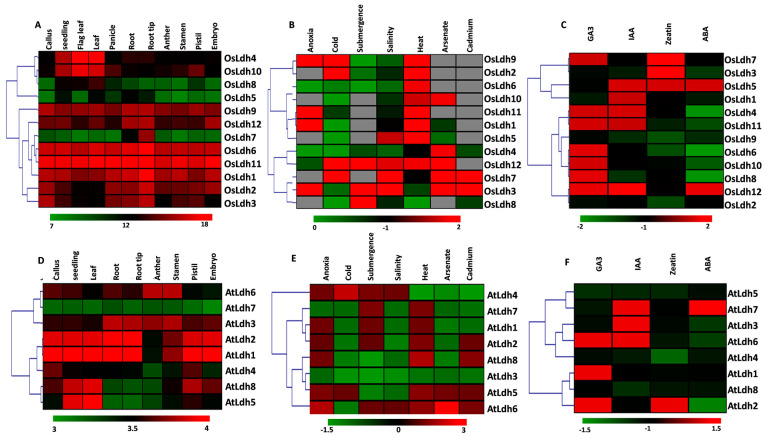
**Tissue–specific and stress induced expression profiling of Ldh transcripts in rice and *Arabidopsis*.** Normalized transcript data of *OsLdh* and *AtLdh* genes were obtained for (**A**,**D**) different tissues and (**B**,**E**) under different stress conditions and (**C**,**F**) hormone treatment as retrieved from the publicly available Genevestigator database. The fold change in expression has been shown as a heatmap generated using the MeV software package. Colour scale below the heatmap shows the level of expression. Hierarchical clustering has been performed on the basis of similarity in expression profiling. Grey colour denotes non-availability of data.

For the study of tissue-specific, stress-mediated and hormone-mediated expression of the *Ldh* genes, normalized expression data of the genes were retrieved from the publicly available Genevestigator database. In rice, *OsLdh7* showed the highest expression (around 16 folds) only in the root tip (Figure 4A). *OsLdh1*, *OsLdh6* and *OsLdh11* were highly expressed in all the tissues, whereas *OsLdh3*, *OsLdh9*, and *OsLdh12* showed the highest expressions in the root and root tip (Figure 4A). Contrastingly, the *Arabidopsis Ldh* genes were found to express constitutively in all the tissues of the plant (Figure 4D). *AtLdh7* showed 3-fold upregulation in the expression in all the tissues of the plant (Figure 4D). Stress-mediated expression profiling of the *Ldh* genes in rice and *Arabidopsis* showed that the members of this family are responsive to a broad range of abiotic stresses. *OsLdh7* and *OsLdh9* showed highest expressions (10-fold) under anoxic conditions (Figure 4B). *OsLdh3* and *OsLdh7* were found to be highly responsive to various heavy metal mediated stresses such as cadmium, arsenate and chromium, respectively, as well as to salinity stress. *OsLdh7*, *OsLdh8*, and *OsLdh12* were found to be highly induced by submergence, whereas *OsLdh9* responded equally to both cold and heat mediated stresses (Figure 4B). The heatmap of the stress-mediated expression profiling of the rice *Ldh* genes indicated a few members such as *OsLdh3*, *OsLdh7*, *OsLdh9*, and *OsLdh12* to be highly responsive to a broad range of abiotic stresses. The *Arabidopsis Ldh* genes, like *AtLdh4*, showed a moderate response to submergence and salinity as well as anoxia. They were found to be highly responsive (3-fold) to cold stress (Figure 4E). *AtLdh6* was found to be highly induced by arsenic stress. *AtLdh1*, *AtLdh2*, and *AtLdh7* were upregulated under submergence conditions (Figure 4E).

A hormone-mediated expression profile showed *OsLdh3*, and *OsLdh7* to be highly responsive to Zeatin (Figure 4C). *AtLdh6* was induced by both IAA and GA, while *AtLdh1* and *AtLdh2* were only GA responsive and *AtLdh3* and *AtLdh7* were both upregulated by IAA solely (Figure 4F). These results suggest that *Ldh* genes are differentially regulated in response to stress and hormone treatments.

### 2.4. Identification of cis-Elements in the Promoter Region of Rice Ldh Genes

Putative cis-elements in the promoter region of rice *Ldh* genes were identified using the PlantCARE database. The analysis shows the presence of various transcription factor binding sites in the 1Kb upstream region of rice *Ldh* genes. These cis-elements identified were found to be involved in growth and development, light and stress response.

**Figure 5:**
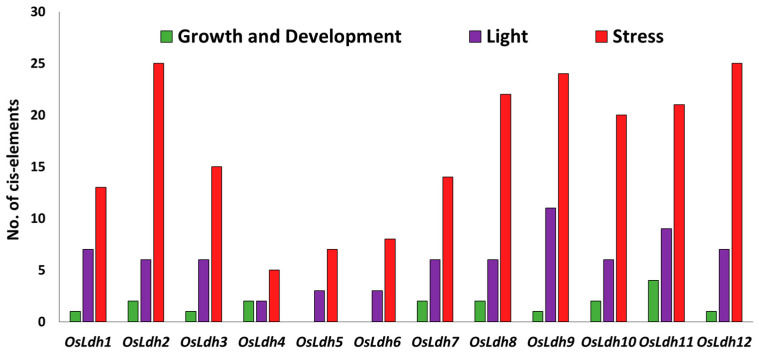
**Identification of the cis-elements in the 1 Kb promoter region of rice *Ldh* genes.** Promoter analysis was carried out to identify the putative cis-elements present in the promoter region of the *Ldh* genes. The cis-elements were identified using the PlantCARE database. The Y-axis denotes the number of cis-elements present in the promoter region of each of the genes, regulating growth and development, light and stress responsiveness. Green colour denotes cis-elements involved in growth and development, purple colour denotes light responsiveness and red colour denotes stress responsiveness.

The list of cis-elements present in the promoter region of each of the *OsLdh* genes has been denoted in Table S1. From Figure 5, it is clear that most of the *OsLdh* genes harbour more than 10 stress-responsive cis-elements such as STRE, CGTCA, ERE, P-BOX, ARE, TC rich repeats, and TGA elements in their promoter region, indicating their putative role in stress response. The highest number of stress responsive cis-elements was present in *OsLdh2* and *OsLdh12*, followed by *OsLdh9. OsLdh7* had the highest number of ABREs, indicating its possible role in drought stress response.

Interestingly, *OsLdh5* and *OsLdh6* had no cis-elements attributed to growth and development, while the other genes had, relatively, a smaller number of cis-elements related to growth and development. *OsLdh8* and *OsLdh12* had the highest number of light-responsive cis-elements, suggesting their putative role in light response.

### 2.5. Temporal Profiling of Rice Ldh Genes under Different Abiotic Stresses Using qRT PCR

**Figure 6:**
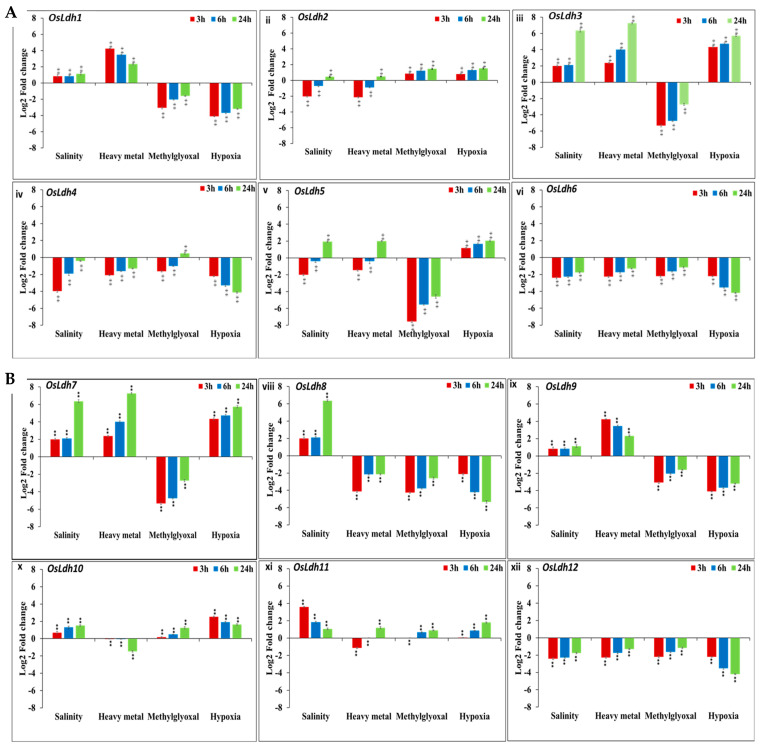
**Transcript abundance analysis of *OsLdh* members under various stress conditions as determined by real time expression analysis.** Bar graphs depicting expression levels of (**A**) (**i**) *OsLdh1*, (**ii**) *OsLdh2*, (**iii**) *OsLdh3*, (**iv**) *OsLdh4*, (**v**) *OsLdh5* and (**vi**) *OsLdh6*; (**B**) (**vii**) *OsLdh7*, (**viii**) *OsLdh8*, (**ix**) *OsLdh9*, (**x**) *OsLdh10*, (**xi**) *OsLdh11* and (**xii**) *OsLdh12* genes under different abiotic stress treatments viz. salinity (200 mM), heavy metal (arsenic, 100 µM), methylglyoxal (10 mM), and hypoxia (given to 21 d old seedlings for 24 h). Expression levels (log2-fold change) have been calculated with respect to the untreated control (having value of 0). Statistical analysis has been performed with three replicates using Student’s *t*-test. ** signifies *p* value < 0.05 up to three or more decimal places and * signifies *p* value < 0.05 for two decimal places.

To validate the influence of stress on the expression of rice *Ldh* genes, we carried out transcript profiling of the genes using qRT PCR in seedlings subjected to different abiotic stresses. The publicly available data suggested a plausible role of few of the genes under oxygen deficit conditions as well as heavy metal stress. To investigate these data, 21-day-old IR64 seedlings were subjected to methylglyoxal (MG), heavy metal (arsenate), hypoxia and salinity stress for time intervals of 3 h, 6 h, and 24 h, described in detail in the material and methods section. Under heavy metal stress, *OsLdh1* was elevated three- to fourfold (Figure 6(Ai)); *OsLdh3* had maximum induction at 3 h, which remained elevated throughout the course of treatment (Figure 6(Aiii)). *OsLdh7* showed maximum upregulation (eightfold) at 24 h (Figure 6(Avii)). The expression of *OsLdh9* peaked at 3 h, gradually decreasing with the increasing time points (Figure 6(Bix)), while *OsLdh10* was downregulated twofold (Figure 6Bx). Under salinity stress, the highest expression was that of *OsLdh7* (sevenfold), followed by *OsLdh1* (fourfold), *OsLdh9* (fourfold) and *OsLdh11* (twofold) (Figure 6(Ai,Bvii,ix,xi)). *OsLdh4* and *OsLdh5* were downregulated at initial time points, but mildly elevated at the 24 h time point. However, *OsLdh2* remained downregulated throughout (Figure 6(Aii,iv,v)). Under methylglyoxal stress, a few genes such as *OsLdh2, OsLdh6*, *OsLdh10* and *OsLdh11* showed upregulation one- to twofold, whereas *OsLdh5, OsLdh8* and *OsLdh9* were highly downregulated (Figure 6(Aii,v,vi,Bviii–xi)). Under hypoxic conditions, *OsLdh3* and *OsLdh7* were upregulated six- and fourfold, respectively, while *OsLdh5, OsLdh10* and *OsLdh11* showed an upregulation of around twofold (Figure 6(Aiii,v,Bvii,x,xi)). On the other hand, *OsLdh4, OsLdh8* and *OsLdh12* were downregulated 4.4 and 6-fold, respectively (Figure 6(Aiv,Bviii,xii)). Thus, from the qRT PCR profiling, it can be concluded that a few members of the rice Ldh gene family, such as *OsLdh3, OsLdh7, OsLdh9* and *OsLdh10*, were highly elevated under stresses such as salinity, hypoxia and heavy metal, suggesting their possible role in combating major abiotic stresses.

### 2.6. Identification of Functionally Active L-Lactate Dehydrogenases Using Schrodinger Suite 

To identify the probable function of the enzymes, it was critical to identify the ligands to which they can bind efficiently. The docking score, RMSD value and interacting amino acids of each of the enzymes with malate and L-lactate have been enlisted in Table 2. The higher the docking score, the higher the probability of binding of the ligand to the protein. Contrastingly, the lower the RMSD value, the greater the stability of the docked complex. 

**Figure 7:**
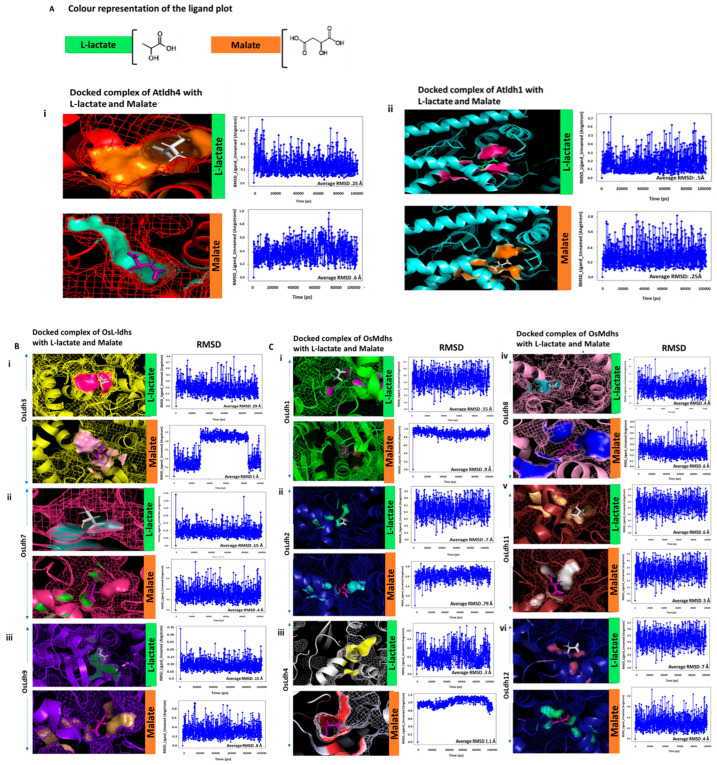

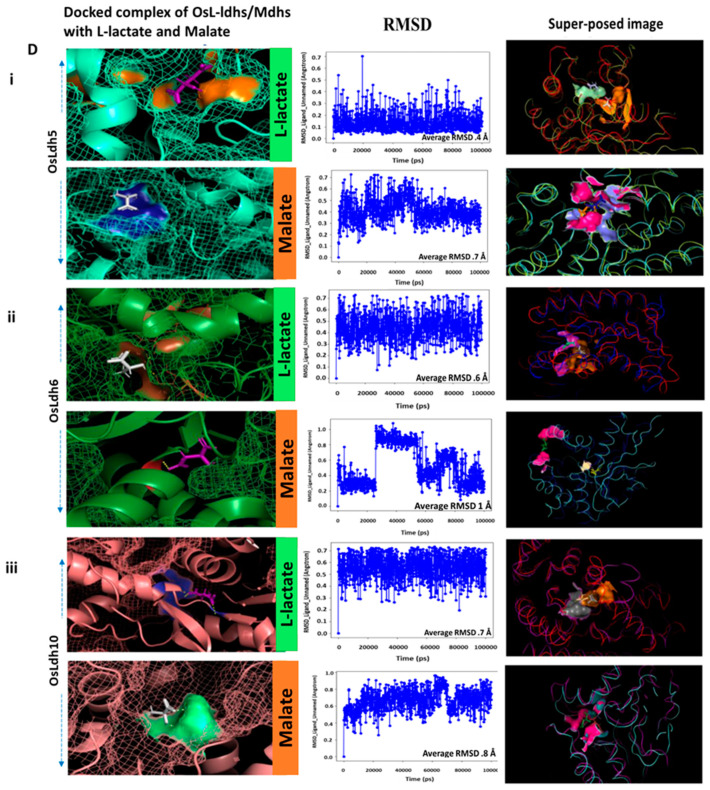
(**A**) **Consolidated colour plot for docked complex of AtLdh1 and AtLdh4 proteins.** (**i**) AtLdh4 docked with L-lactate and malate. (**ii**) AtLdh1 docked with L-lactate and malate. The RMSD graphs of each of the proteins have also been plotted. Y-axis denotes RMSD value (Å) and X-axis denotes time (ps). The ligands, L-lactate (green) and malate (orange), have been depicted with colour strips on the right side of the figures. Docking has been carried out using Schrodinger Suite. The average RMSD value has been mentioned in each of the graphs. (**B**) **Putatively functional L-lactate dehydrogenases in rice**. (**i**) OsLdh3, (**ii**) OsLdh7 and (**iii**) OsLdh9 docked with L-lactate (green) and malate (orange). The RMSD graph of each of the proteins have also been plotted. (**C**) **Putatively functional malate dehydrogenases in rice**. (**i**) OsLdh1, (**ii**) OsLdh2 (**iii**) OsLdh4, (**iv**) OsLdh8, (**v**) OsLdh11 and (**vi**) OsLdh12 docked with L-lactate (green) and malate (orange). The RMSD graphs of each of the proteins have also been plotted and depicted. Y-axis denotes RMSD value (Å) and X-axis denotes time (ps). Docking has been carried out using Schrodinger Suite. The average RMSD value has been mentioned in each of the graphs. (**D**) **Putatively functional malate/L-lactate dehydrogenases in rice**. (**i**) OsLdh5, (**ii**) OsLdh6 and (**iii**) OsLdh10 docked with L-lactate (green) and malate (orange). The RMSD graphs of each of the proteins have also been plotted. Y-axis denotes RMSD value (Å) and X-axis denotes time (ps). The respective proteins were super-posed with the enzymatically active AtLdh1 (cyan blue) and AtLdh4 (red) to depict the orientation of the active site geometry. Docking has been carried out using Schrodinger Suite. The average RMSD value has been mentioned in each of the graphs.

The AtLdh4 and AtLdh1 enzymes have been functionally reported as L-lactate dehydrogenase and malate dehydrogenase, respectively, in *Arabidopsis*. With these two as reference proteins, the aim of this study was to determine the putative functionally active L-lactate dehydrogenase in rice, keeping the RMSD value and docking score as the selection parameters. The AtLdh4 protein was docked with L-lactate and malate separately. The docking score with L-lactate was −4.67, while with malate was −4.304. The RMSD values of the docked complexes were 0.25 Å and 0.5 Å, respectively. Considering the parameters, it could be deduced that the AtLdh4–lactate complex was more stable than the AtLdh4–malate complex (Figure 7(Ai)). Similarly, the AtLdh1 protein when docked with L-lactate and malate had a docking score of -6.98 for malate and −3.45 for L-lactate. The RMSD for the AtLdh1–malate complex was 0.25 Å, while for AtLdh1–L-lactate it was 0.6 Å, indicating the AtLdh1–malate complex to be more stable and favourable (Figure 7(Aii)). OsLdh3, Osldh7 and OsLdh9 had docking scores of −3.864, −6.693 and −4.042 when docked with L-lactate. OsLdh3, OsLdh7, and OsLdh9 had docking score of −3.4, −2.4 and −1.865 when docked with malate (Figure 7(Bi–iii)). The RMSD values were 0.61 Å, 0.9 Å and 0.8 Å, respectively. These results suggest that these three proteins had a greater affinity towards L-lactate than malate, and they formed stable docked complexes with lactate as a ligand. On the other hand, OsLdh11 and OsLdh12 both had a docking score of −4.634 when docked with malate and that of −3.619 when docked with lactate (Figure 7(Cv,vi)). The RMSD values of the malate-docked complex of OsLdh11 and OsLdh12 were 0.5 Å and 0.4 Å, while those of the lactate-docked complex of OsLdh11 and OsLdh12 were 0.6 Å and 0.7 Å, respectively. This suggested that these enzymes were plausibly malate dehydrogenases. Interestingly, though the RMSD value for OsLdh1, OsLdh2, OsLdh4, and OsLdh8 docked with malate was more than that of L-lactate, yet the RMSD was much more stable throughout the 100 ns simulation compared to that when bound with L-lactate. (Figure 7(Ci–iv)). OsLdh10 had a docking score of −3.167 and −3.253 and RMSD value of 0.8 Å and 0.7 Å when docked with malate and lactate, respectively. Similarly, OsLdh5 and OsLdh6 had a docking score more or less similar for both the ligands. Since from these parameters it was difficult to draw conclusions about their ligand affinity, it was important to ascertain the similarity of the site geometry confirmation of these proteins with either of the functionally active lactate/malate dehydrogenases. Therefore, the homology modelling and super-position of the modelled proteins was carried out with previously characterized AtLdh4 and AtLdh1 proteins. The active site geometry of OsLdh5 and OsLdh10 were similar to that of AtLdh1. This indicated that, although both these proteins were structurally aligned to functionally active L-lactate dehydrogenase (AtLdh4), they may be putatively functional malate dehydrogenases based on the similarity of their site geometry (Figure 7(Di,iii)). OsLdh6, on the other hand, could be putatively functional L-Ldh as both its structure and active site geometry closely resemble functional AtLdh4. Additionally, it formed a more stable complex with lactate as compared to malate (Figure 7(Dii)). Thus, we conclude that the putatively functional L-lactate dehydrogenases in rice are OsLdh3, OsLdh7 and OsLdh9. Moreover, we found that the active site arrangement of OsLdh3 and OsLdh7 enzymes shared more similarity with functionally active AtLdh as compared to OsLdh9. The common interacting amino acids included Ser-219 and Glycine-220. The plausibly functional malate dehydrogenases, OsLdh11 and OsLdh12, shared a similar active site arrangement with AtLdh1. The common interacting amino acids were Asn and Arg. The interacting amino acids in the active site geometry of each of the Ldh proteins when docked with ligands L-lactate and malate are shown in Figures S1 and S2.

## 3. Discussion

The Ldh/Maldh enzymes belong to a superfamily, having a common evolutionary origin. Genome-wide identification of malate dehydrogenases in cotton [20], apple [21] and poplar [22] reveals it to be a multigene family involved in fibre development, organic acid metabolism and salinity stress response, respectively. Although the presence of L-lactate dehydrogenases has been experimentally proven in tomato [18], barley [16], soybean [23], maize [17] and *Arabidopsis* [19], there is no published report on the presence of Ldhs in rice so far. So, in this study, we aimed to comprehensively investigate the functionally active Ldh enzymes in model plants, rice and *Arabidopsis*. Like many other gene families in plants, we found Ldh to be a multigene family, consisting of twelve and eight members in rice and *Arabidopsis*, respectively. Evolutionary analysis revealed a clustering of lactate dehydrogenases into different clades consisting of proteins from either the same or different species, indicating diversification. However, the putatively functional OsLdh proteins clustered with the previously characterized functional AtLdh4 protein suggests a slower rate of mutation in the functional region of the protein. The majority of the OsLdh and AtLdh proteins localized in the cytoplasm, while others localized in the mitochondria and chloroplast. In an earlier study, Ldh isolated from leaves of lettuce plants was proposed to be involved in the regulation of cellular pH and maintaining the reducing equivalents in the leaf cytoplasm [14]. Thus, the presence of AtLdh4 and OsLdh3/7 in cytoplasm indicates their possible role in controlling cellular pH and the level of cellular acidity, implying involvement in cellular acidosis. Ldh enzymes isolated from the mitochondria of potato tubers have been shown to be involved in the conversion of lactate to pyruvate. Under oxygen deficit conditions, a decrease in mitochondrial respiration leads mitochondrial Ldh to play an important role in plant adaptation to hypoxia [24]. It could, therefore, be predicted that mitochondrial localized OsLdh6 and AtLdh2/3 may have a similar role under oxygen deficit conditions in rice. Likewise, OsLdh2/4 and AtLdh8, predicted to be malate dehydrogenases, are found to be localized in the chloroplast, where they are presumed to be involved in the photosynthetic pathways. Maldh are reported to be involved in the biosynthesis of malic acid in cytosol [25]. Therefore, it can be assumed that the predicted cytosolic malate dehydrogenases, OsLdh11 and OsLdh12, might have a similar role. 

Previous studies have shown *Ldh* genes to have an established role under oxygen deficit conditions [16,17,18,26]. However, their role in other abiotic stresses still remain elusive. The 1Kb upstream sequence of each of the *Ldh* genes harboured different abiotic stress responsive elements, thereby suggesting their role and regulation under different stresses. Previously identified *AtLdh4* is the only *Ldh* gene known to be involved in other abiotic stresses such as drought, cold and wounding [19]. This interested us in the study of the abiotic stress-mediated regulations of *Ldh* genes in rice and Arabidopsis. A detailed analysis of publicly available data, as well as real time expression profiling, shows the differential regulation of the genes under various abiotic stresses. The Genevestigator data showed *OsLdh3* and *OsLdh7* to be highly regulated by the phytohormone zeatin. Zeatin, along with other plant hormones, is reported to alleviate heavy metal stress mediated symptoms in lower group of plants by impeding heavy metal absorption, thus reducing oxidative stress caused by lipid peroxidation and hydrogen peroxide levels [27,28]. Since *OsLdh3* and *OsLdh7*, predicted to be localized in the root tip, showed remarkable responses under heavy metal inflicted stress conditions, it can be assumed that zeatin might reduce heavy metal mediated phytotoxicity in rice by regulating these Ldh enzymes, much like the two-component system, specifically histidine kinases, which mediate cross-talk between hormone and stress responsive cascades through their roles as osmosensors [29,30]. In the case of salinity stress, *OsLdh3*, *OsLdh7* and *OsLdh9* show marked up-regulation, similar to the animal systems [31,32]. 

Methylglyoxal (MG), which is a possible biomarker of plant stress response [33], is yet another source for the generation of L-lactate in the plants via the action of methylglyoxal reductases (MGR) and lactaldehyde [34]. Thus, we were interested to study the effect of MG on the transcript abundance of *Ldh* genes in rice. The exogenous application of MG resulted in a 2–2.5-fold induction of *OsLdh6*, *OsLdh9*, *OsLdh10*, thereby indicating lactate accumulation upon MG treatment. Malate dehydrogenase and lactate dehydrogenase enzymes, owing to their homology, share a common protein fold and catalytic mechanism. Yet, they are known to have a strict capacity to differentiate between their substrates [2]. The essential amino acids required for substrate specificity and catalysis have been identified through crystallographic and site-directed mutagenesis studies for the forward reaction [35,36,37]. However, no such information about substrate specificity is known, especially in plants, to the best of our knowledge, during the reverse reactions, i.e., from malate/lactate to oxaloacetate/pyruvate. Therefore, to identify the interacting amino acids involved in binding the respective ligands with each of the proteins, and to determine the stability of the complex so formed, we carried out a docking analysis using Schrodinger Suite. The docking score, RMSD value and stability of the RMSD throughout the simulation of 100 ns are the critical criteria to distinguish the L-lactate dehydrogenases (L-Ldh) from the malate dehydrogenases. The overall analysis gave insights to the putative functional OsL-Ldh such as OsLdh3, OsLdh7 and OsLdh9, and OsMaldh such as OsLdh11 and OsLdh12. As discussed before, the functionally active AtLdh4 protein had a docking score of −4.67 when docked with L-lactate and −4.304 when docked with malate. The average RMSD value of the lactate–AtLdh4 complex was 0.25, while that for the malate–AtLdh4 complex was 0.6. Clearly, the result indicated that AtLdh4 has better binding affinity with L-lactate than malate. The common interacting amino acids for AtLdh4, OsLdh3 and OsLdh7 were Ser-219 and Gly-220. This suggests that perhaps these two amino acids might have a role in recognizing L-lactate as a ligand, though this can be only concluded by carrying out further experiments. On the other hand, OsLdh9 also had a stable RMSD value of 0.15 Å throughout the simulation period when docked with L-lactate, but the interacting amino acids His-251 and Arg-227 were not similar to that of AtLdh4 protein, possibly the reason it was not designated as a L-ldh in the Prosite scan. However, it is reported that histidine does have a role in the molecular functioning of the Ldh enzyme [38]. Though histidine does not directly interact with AtLdh4 when docked with ligand L-lactate, it is present in the active site orientation of the docked complex. Thus, it can be assumed that, along with Ser-219 and Gly-220, His-251 might also have a role in L-lactate dehydrogenase enzyme activity. Similarly, in the case of malate dehydrogenase enzymes, the putatively functional ones such as OsLdh11 and OsLdh12 shared common interacting amino acids, An-193, Val-195 and Arg-227, with functionally active malate dehydrogenase, AtLdh1. In the case of the other predicted malate dehydrogenases, OsLdh2 had common interacting amino acids with OsLdh11/12 when docked with malate, while OsLdh4 and OsLdh8 had Asn in common with OsLdh11/12, though the position of the respective amino acids was different. These results indicate that Asn and Arg might have a role to play in malate dehydrogenase activity. Reports suggest the existence of L-lactate such as malate dehydrogenase enzymes in the prokaryotic kingdom [39,40]. These enzymes are structurally similar to L-lactate dehydrogenases, but functionally similar to malate dehydrogenases. In this context, we found that although OsLdh5 and OsLdh10 had similar structural alignment to AtLdh4, their active site geometry was dissimilar to that of AtLdh4. These proteins, when superposed with AtLdh1, showed similar active site geometry to the latter. Thus, it can be predicted that these proteins might have structural similarity to the active L-lactate dehydrogenase enzymes but functionally they might be malate dehydrogenases. OsLdh6, on the other hand, had extremely dissimilar active site geometry with the functional malate dehydrogenase but shared similar site geometry to that of AtLdh4, citing that it might have binding affinity to L-lactate more than malate as a ligand. These conclusions from the in silico analysis can be authenticated only after the functional validation of these proteins. 

Nevertheless, the overall study helped us to identify the putatively functional L-ldh in rice amongst the twelve-member family. As determined by the Schrodinger analysis, OsLdh3, OsLdh7 and OsLdh9 have been found to be putatively functional Ldhs in rice. The expression levels of these three Ldhs are also highly upregulated under various stresses, especially salinity, hypoxia and heavy metal mediated stresses. *OsLdh3* and *OsLdh9* are found to express uniformly in all the different tissues, and *OsLdh7* specifically in the root. Among the three putatively functional OsLdhs, OsLdh7 has the highest docking score (−6.693) and the lowest RMSD value (0.15). Thus, based on our analysis, it is highly likely that OsLdh7 is the candidate most likely to be functioning as Ldh, regulating the edaphic stress factors such as heavy metal, salinity and hypoxia, and conferring stress tolerance to plants. Therefore, we propose these enzymes to have a convincing role in combating major abiotic stresses such as heavy metal, hypoxia and salinity by mediating the reversible reaction of lactate to pyruvate, thus controlling cellular acidosis and its detrimental effects on plants.

## 4. Materials and Methods

### 4.1. Identification, Characterization and Domain Assessment of Ldh Genes/Proteins across Plant Species

Rice *Ldh* genes were identified using the previously characterized Ldh protein sequence of Arabidopsis [19]. For this, a BlastP search was conducted in RGAP (http://rice.uga.edu, accessed on 15 January 2021) and TAIR (www.arabidopsis.org, accessed on 18 January 2021) with e-value threshold ≤ 10^−3^. Thereafter, the domain architecture was drawn manually. Physical properties of protein, such as polypeptide length, pI, and molecular weight, were predicted using the ExPasyProtParam (https://web.expasy.org/protparam/, accessed on 1 February 2021) tool. Chromosomal location, gene length, and CDS coordinate (5′ to 3′) were retrieved from the Phytozome database. Subcellular localizations of each Ldh protein were predicted using DeepLoc (http://www.cbs.dtu.dk/services/DeepLoc/, accessed 5 March 2021) [41]. Chloroplast localization was confirmed using ChloroP software (http://www.cbs.dtu.dk/services/ChloroP/, accessed on 5 March 2021). The presence of signature active sites for Ldh (PS00064) and Maldh (PS00067) were confirmed using PROSITE (http://prosite.expasy.org/, accessed on 23 July 2021). 

### 4.2. Evolutionary Analysis of Genes Encoding Ldh across Plant Species 

Multiple sequence alignment of the Ldh members, derived from *Arabidopsis thaliana* (8 proteins) and *Oryza sativa* (12 proteins), were performed using ClustalΩ (https://www.ebi.ac.uk/Tools/msa/clustalo/, accessed on 21 January 2021) with default parameters. The alignment result was used for the evolutionary analysis. The phylogenetic tree was constructed using the Neighbour-joining method. The tree was visualized using the Itol software (https://itol.embl.de, accessed on 23 May 2021).

### 4.3. Gene Structure Analysis and Motif Identification of the Ldh Genes/Proteins 

The illustration of *Ldh* gene structures was analysed using the Gene Structure Display Server 2.0 (http://gsds.cbi.pku.edu.cn/, accessed on 15 February 2021). Conserved motifs in the putative OsLdh and AtLdh protein family were predicted using the Multiple Expectation Maximization for Motif Elicitation (MEME) program (http://meme-suite.org/, accessed on 20 February 2021) with the default parameters and the maximum number of motifs was set as 10. 

### 4.4. Identification of Putative cis-Regulatory Elements in the Promoter Region of Rice Ldh

The 1000 bp of 5′ upstream DNA sequences of all the *OsLdh* genes were retrieved from the RGAP database and analysed using the PlantCARE database (http://bioinformatics.psb.ugent.be/webtools/plantcare/html/, accessed on 22 February 2021) for the prediction of putative hormone or stress-responsive cis-regulatory elements.

### 4.5. Tissue-Specific and Stress Mediated Expression Profiling of Ldh Proteins in Rice and Arabidopsis

The expression profile of *OsLdh* and *AtLdh* genes under different abiotic stress conditions such as, anoxia, cold, submergence, salinity, heavy metal (chromium, cadmium and arsenate) hormonal treatments and various tissue-specific stages were retrieved from the Genevestigator database (https://genevestigator.com, accessed on 23 March 2021). Heat maps were generated and hierarchical clustering was carried out using the MeV software package [42]. 

### 4.6. Plant Materials and Stress Treatments

To study the expression profile of the *Ldh* genes, IR64 rice (Oryza sativa cv. indica) seeds supplemented with Yoshida media were grown in sterile germination rolls under controlled conditions in the growth chamber at 28 °C for 21 days. Seedlings were then subjected to heavy metal (100 μM arsenate), methylglyoxal (10 mM), salinity (200 mM NaCl) and hypoxia stress treatments under controlled conditions. The samples were harvested at 3, 6, 24 h. Untreated samples served as a control.

### 4.7. RNA Isolation, cDNA Synthesis and qRT PCR Analysis of Rice Ldh Genes

Total RNA was isolated using TRIzol™ reagent (Sigma Aldrich, Burlington, MA, USA) as per the manufacturer’s protocol. First strand cDNA was synthesized using RevertAid first strand cDNA synthesis kit (Thermo Fischer Scientific, Waltham, MA, USA). Primers used for the experiment are listed in Table S2. The qRT-PCR was performed, and the specificity of the amplification was tested by melt curve analysis. Three technical replicates were analysed for each sample. The Log2 fold change values of each of the candidate genes were calculated using the delta Ct value method [43]. Normalization of the transcript level of each gene in different samples was carried out with respect to the internal control gene, elongation factor 1-alpha (EF-1a). The statistical analysis for three replicates was performed using Student’s *t*-Test (*p* value < 0.05).

### 4.8. Protein Modelling with Schrodinger Suite

The modelling of protein sequences of AtLdh and OsLdh proteins was performed using Schrodinger Suite, Prime module by threading specific energy-based calculations [44,45]. Templates of each protein involved in the model were observed to have more than 60% query coverage and similarity index for each target protein. Structure validation of each modelled protein was carried out by subjecting them to simulation in the water environment for 20 nanoseconds [46]. Behavioural stability of the modelled proteins was performed by Molecular Dynamics Simulation techniques, following which interaction between each protein and ligand candidates malate and L-lactate was calculated by using Glide module from the Schrodinger Suite [47]. The final docking score was reported after post-docking minimization of each complex. Homology modelling was carried out using Prime modelling Suite; proteins were superimposed and visualized in PyMol.

## Figures and Tables

**Table 1:** **Details of putative Ldh genes predicted in *Arabidopsis* and rice genome.** Table enlists Ldh genes in rice and *Arabidopsis*, along with their existing locus identifiers, nomenclature, chromosomal locations, CDS, protein length and sub-cellular localization.

Gene Name	Locus ID	Transcripts	Coordinate (5′-3′)	CDS Length (bp)	Protein			Localisation
					Amino Acid Length	pI	Mol. wt (kDa)	
*AtLdh1*	AT1G04410	AT1G04410.1	1189078-1191412	999	332	6.11	35.57	cytoplasm
*AtLdh2*	AT1G53240	AT1G53240.1	19854615-19856937	1026	341	8.54	35.8	mitochondria
*AtLdh3.1*	AT3G15020	AT3G15020.1	5056068-5058248	1026	341	8.3	35.87	mitochondria
*AtLdh3.2*		AT3G15020.2	5056068-5058248	951	316	9.49	33.13	mitochondria
*AtLdh4*	AT4G17260	AT4G17260.1	9673991-9675448	1062	353	6.07	37.95	cytoplasm
*AtLdh5.1*	AT5G09660	AT5G09660.1	2993444-2995675	1065	354	8.14	37.36	cytoplasm
*AtLdh5.2*		AT5G09660.2	2993444-2995307	1002	333	7.56	34.95	cytoplasm
*AtLdh5.3*		AT5G09660.3	2993444-2995307	1029	342	9.06	36.34	cytoplasm
*AtLdh5.4*	AT5G09660	AT5G09660.4	2993444-2995675	1092	363	9.16	38.73	cytoplasm
*AtLdh6*	AT5G43330	AT5G43330.1	17390433-17392645	999	332	6.33	35.67	cytoplasm
*AtLdh7*	AT5G56720	AT5G56720.1	22945537-22946718	1020	339	5.75	36.87	cytoplasm
*AtLdh8.1*	AT5G58330	AT5G58330.1	3579722-23582395	1332	443	5.81	48.3	chloroplast
*AtLdh8.2*		AT5G58330.2	23579722-23582395	1329	442	5.81	48.22	chloroplast
*AtLdh8.3*		AT5G58330.3	23579722-23582295	1005	334	4.95	36.39	cytoplasm
*OsLdh1*	Os01g46070	Os01g46070.1	26190752-26194517	1023	340	8.74	35.46	mitochondria
*OsLdh2*	Os01g61380	Os01g61380.1	35499017-35501765	1191	396	7.63	41.78	chloroplast
*OsLdh3*	Os02g01510	Os02g01510.1	295302-299174	1179	392	6.74	42.71	cytoplasm
*OsLdh4*	Os03g56280	Os03g56280.1	32089685-32086001	1065	354	8.13	37.02	cytoplasm
*OsLdh5*	Os04g46560	Os04g46560.1	27605166-27608347	1059	353	6.72	38.29	mitochondria
*OsLdh6*	Os05g49880	Os05g49880.1	28621585-28617595	1023	340	8.22	35.43	mitochondria
*OsLdh6*	Os05g49880	Os05g49880.2	28621585-28617595	696	231	7.21	32.93	mitochondria
*OsLdh7*	Os06g01590	Os06g01590.1	348516-346985	1083	360	7.9	38.72	cytoplasm
*OsLdh8*	Os07g43700	Os07g43700.1	26155933-26153825	1215	404	9	42.22	cytoplasm
*OsLdh9*	Os08g33720	Os08g33720.1	21057561-21054659	1194	397	7.02	41.53	chloroplast
*OsLdh10*	Os08g44810	Os08g44810.1	28141042-28146270	1302	434	6.96	47	chloroplast
*OsLdh11*	Os10g33800	Os10g33800.1	17913818-17917850	999	333	5.75	35.56	cytoplasm
*OsLdh12*	Os12g43630	Os12g43630.1	27099351-27094647	1071	357	8.09	37.38	chloroplast

At: Arabidopsis thaliana; Os: Oryza sativa.

**Table 2:** The docking score, RMSD value and interacting amino acids of each of the putative Ldh enzymes with malate and L-lactate as ligands are enlisted in the table.

Proteins	Ligand					
	Malate			L-Lactate		
	Interacting Amino Acids	Docking Score	RMSD Value (Å)	Interacting Amino Acids	Docking Score	RMSD Value (Å)
AtLdh1	Asn-193	−6.993	0.25	Arg-227	−4.3	0.5
	Val-195	−6.993		His-251	−4.3	
	Arg-227	−6.993				
AtLdh4	Ser-195	−4.304	0.6	Ser-219	−4.67	0.25
	Ser-219			Gly-220		
	Leu-223			Leu-223		
	His-251			Ser-313		
	Ser-313					
Osldh1	Thr-222	−4.384	0.9	Arg-227	−3.619	0.55
				His-251		
OsLdh2	Asn-193	−4.249	0.79	Arg-227	−3.619	0.7
	Arg-227			His-251		
OsLdh3	Leu-223	−3.443	0.6-1	Asn-196	−3.864	0.29
	Asp-224			Ser-219		
				Gly-220		
OsLdh4	Ile-158	−4.234	1.1	Asn-160	−4.205	0.3
	Asn-160			Val-187		
	Leu-190			Leu-190		
OsLdh5	Asn-193	−4.634	0.7	Val-151	−3.325	0.4
	Val-195			Asn-153		
	Arg-227					
OsLdh6	Thr-222	−3.619	1	Arg-227	−3.619	0.6
				His-251		
OsLdh7	Asn-193	−2.4	0.4	Ser-219	−6.693	0.15
	Val-195			Gly-220		
	Arg-227					
OsLdh8	Arg-157	−3.489	0.6	Val-222	−3.258	0.4
	Asn-195			Leu-225		
	Arg-229			Asp-226		
OsLdh9	Asn-193	−1.865	0.8	Arg-227	−4.042	0.15
	Val-195			His-251		
	Arg-227					
OsLdh10	Leu-159	−3.167	0.8	Val-130	−3.253	0.7
OsLdh11	Asn-193	−4.634	0.5	Arg-227	−3.619	0.6
	Val-195			His-251		
	Arg-227					
OsLdh12	Asn-193	−4.634	0.4	Arg-227	−3.619	0.7
	Val-195			His-251		
	Arg-227					

## Data Availability

The datasets supporting the conclusions of this article are included within the article and its additional files. The sequence data obtained for rice and *Arabidopsis* were retrieved from the RGAP (http://rice.plantbiology.msu.edu/, accessed on 26 December 2022) and TAIR (https://www.arabidopsis.org/, accessed on 26 December 2022) databases, respectively.

## Supplementary Materials

The following supporting information can be downloaded at: https://www.mdpi.com/article/10.3390/ijms24065900/s1.
