# Supplementary material for: Lactate Dehydrogenase Superfamily in Rice and Arabidopsis: Understanding the Molecular Evolution and Structural Diversity
Source: Int J Mol Sci. 2023 Mar 21;24(6):5900. doi: 10.3390/ijms24065900 (PMC10057475; doi:10.3390/ijms24065900)
Supplement: Supplementary file 1 [file ijms-24-05900-s001.zip › ijms-2156473-supplementary.pdf]

Table S1. List of identified cis elements in the promoter region of rice Ldh genes.

Motifs	Functions
CAT-box	Growth and development
AC-II	Growth and development
CARE	GA induced expression of hydrolase genes in germination
O2-site	Growth and development
GCN4_motif	Growth and development
CCGTCC motif	Growth and development
RY-element	Growth and development
AC-I	Growth and development
HD-Zip 1	Regulate plant growth adaptation responses
G-Box	Light responsive
AE-box	Light responsive
GT1-motif	Light responsive
TCCC-motif	Light responsive
chs-CMA2a	Light responsive
Box4	Light responsive
A-box	Elicitor, Light responsive
ACE	Light responsive
Sp1	Light responsive
GC-motif	Light responsive
GATA-motif	Light responsive
TCT-motif	Light responsive
ATC-motif	Light responsive
Gap-box	Light responsive
3-AF1 binding site	Light responsive
I-box	Light responsive
GA-motif	Light responsive
as-1	biotic stress response
ABRE	ABA regulated gene expression
MYC	Jasmonic acid responsive element
LTR	Low temperature responsive
MBS	Stress responsive
ABRE4	ABA regulated gene expression
ABRE3a	ABA regulated gene expression
DRE Core	Stress responsive (cold and dehydration)
TGACG-motif	Me-JA responsive element, SA responsive element
WRE3	Wound responsive
CGTA-motif	Stress responsive
STRE	Defence and stress related
ERE	Stress responsive
P-box	Stress responsive
TC-rich element	Stress responsive
ARE	Stress responsive
TCA-element	Stress responsive
AuxRR-core	Stress responsive
A-box	Elicitor, Light responsive
W-box	Fungal elicitor, wound responsive

CCGTCC-box	Same as A-box
Wyn-motif	Stress responsive

Table S2. List of primers used for the qRT-PCR study.

Primer	Sequence
OsLdh1_F	AGGCCATTTTGCAGACTAGC
OsLdh1_R	AAGCTCAAATCCTCGGTATCCC
OsLdh2_F	GAAGGCGGAATTTTTCGTGTC
OsLdh2_R	AGGCACAAGGGTCAAATAGC
OsLdh3_F	TTATCATCGTCGTCGTCGTCTC
OsLdh3_R	CATACTACAGTCGAGTCGATCAC
OsLdh4_F	CGACCGTGAAATGCTAATTACCC
OsLdh4_R	GCATGCAAAATTGTTCACAACAG
OsLdh5_F	ATCGTTGCAAGGGTTTCGTC
OsLdh5_R	TCATGAGATGCACCGTGATAGC
OsLdh6_F	CGAGATGTTGTGCTCCAGAA
OsLdh6_R	GGGTCATGATTTGGGTTCAC
OsLdh7_F	CGCCAACCCGTTTATATATGCC
OsLdh7_R	GCGGCCATGAAATTAAGCTTCG
OsLdh8_F	TGCAGTGGTGCTCCATTAGT
OsLdh8_R	GGCAATCCAGTCAGCATTTT
OsLdh9_F	TGCATTGTATTGCCATTGCT
OsLdh9_R	TTGTCCAGCTACAAACCAACC
OsLdh10_F	TTGCCATAGATGCACATGCC
OsLdh10_R	TGGCTGCAACACAATAACCG
OsLdh11_F	TAAACAGCCTCAGCCCTTGT
OsLdh11_R	AGTACTGCGTGGTGGCTTCT
OsLdh12_F	TCTTGGAATGCCCCAATTTA
OsLdh12_R	TCAGAGCAGGGTTCCTTTTT

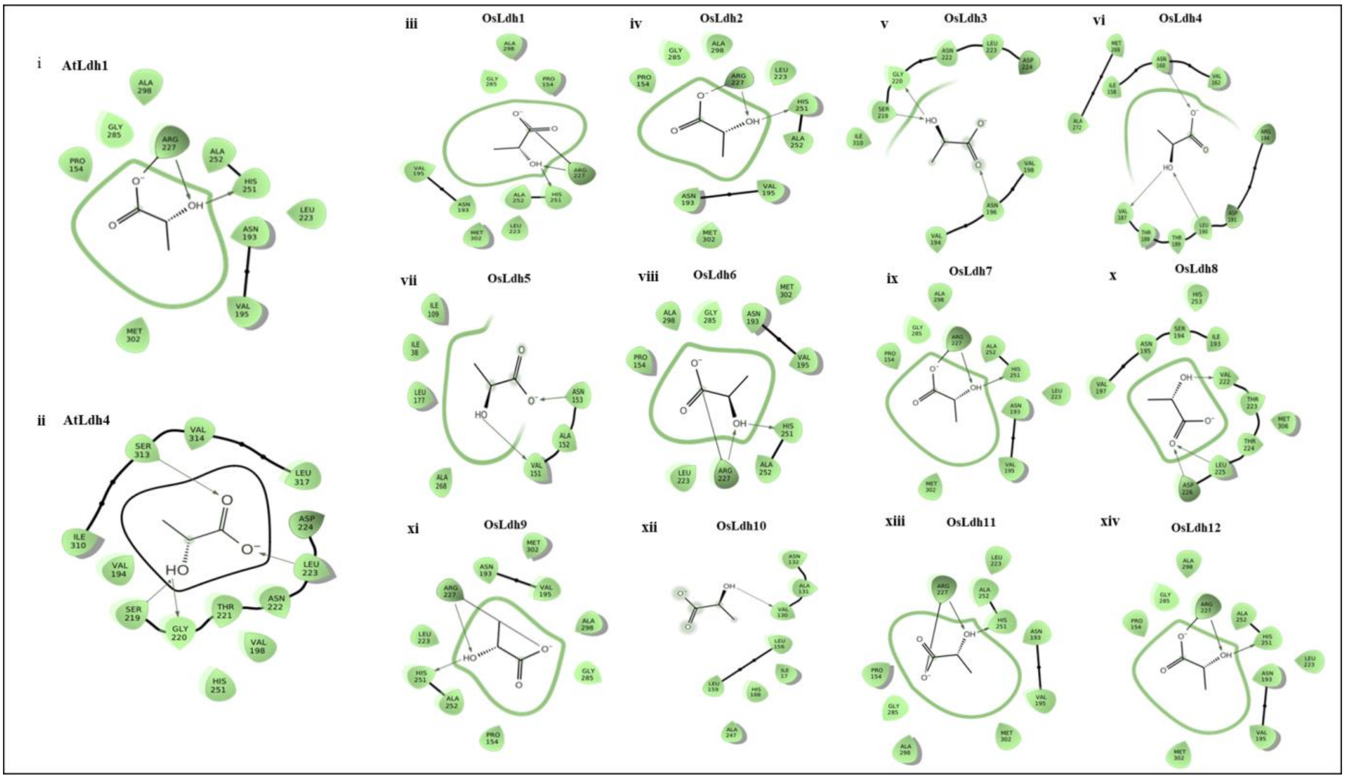

Figure S1. The 2D plot of AtLdh-L-lactate and OsLdh-L-lactate complexes: (i) AtLdh1, (ii) AtLdh4, (iii) OsLdh1, (iv) OsLdh2, (v) OsLdh3, (vi) OsLdh4, (vii) OsLdh5, (viii) OsLdh6, (ix) OsLdh7, (x) OsLdh8, (xi) OsLdh9, (xii) OsLdh10, (xiii) OsLdh11, (xiv) OsLdh12 shows the 2D sketcher plot of the active site geometry of the respective proteins when docked with **ligand L-lactate**. The molecular docking is carried out by the Glide ligand docking module of the Schrodinger Suite. The images have been retrieved from the ligand preparation and library design module of the Schrodinger Suite.

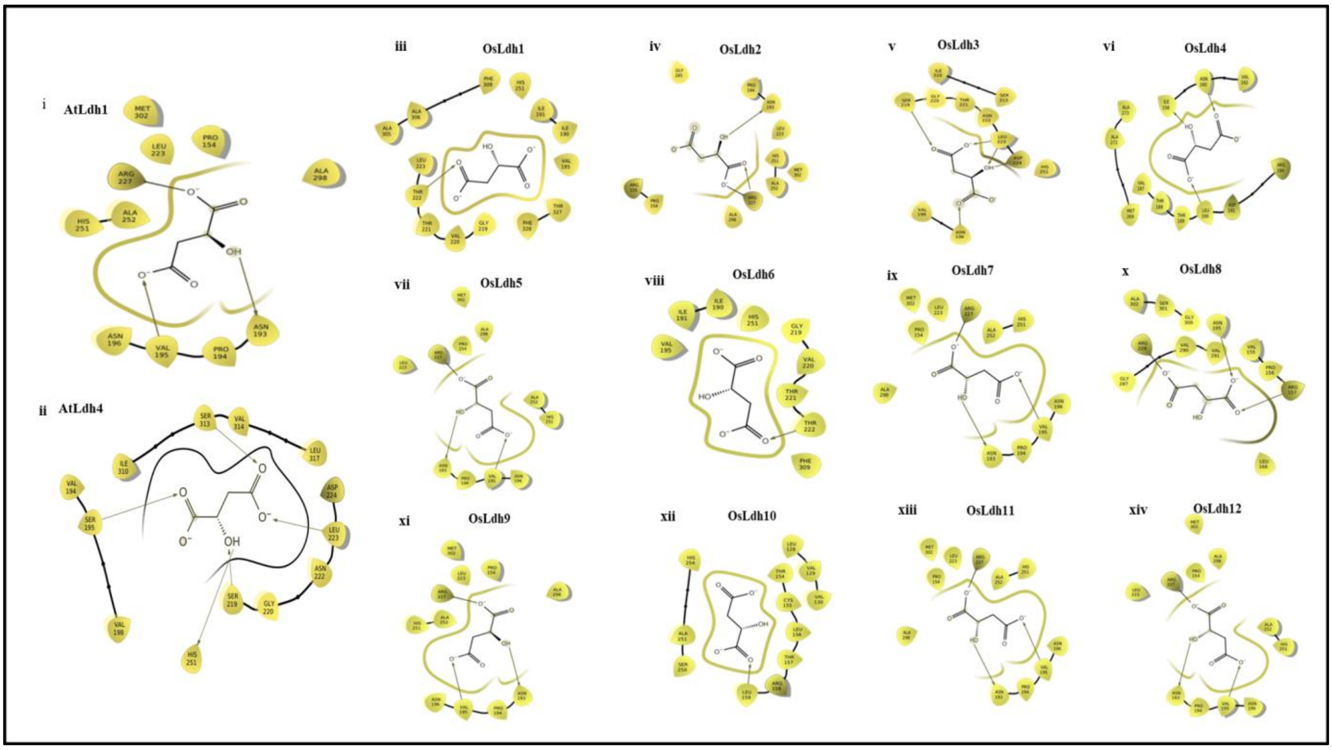

Figure S2. The 2D plot of AtLdh-L-lactate and OsLdh-L-lactate complexes: (i) AtLdh1, (ii) AtLdh4, (iii) OsLdh1, (iv) OsLdh2, (v) OsLdh3, (vi) OsLdh4, (vii) OsLdh5, (viii) OsLdh6, (ix) OsLdh7, (x) OsLdh8, (xi) OsLdh9, (xii) OsLdh10, (xiii) OsLdh11, (xiv) OsLdh12 shows the 2D sketcher plot of the active site geometry of the respective proteins when docked with **ligand malate**. The molecular docking is carried out by the Glide ligand docking module of the Schrodinger Suite. The images have been retrieved from the ligand preparation and library design module of the Schrodinger Suite.
